# Is LOE test useful to recognize LCA insufficiency?

**DOI:** 10.1007/s10195-013-0285-4

**Published:** 2014-01-31

**Authors:** Giuseppe Laurà

**Affiliations:** Knee Surgery Department, Istituto Clinico Città Studi, Milan, Italy

Dear Sir,

“I read the article “The loss of extension test (LOE test): a new clinical sign for the anterior cruciate ligament insufficient knee” by Salvi et al. [1]”, and I think all that is practiced and described could be scarcely suitable for the diagnosis of ACL tear.

I completely agree with the AA on the validity of the knee joint extension maneuver (recurvatum test) as an initial clinical approach in order to test the ACL, but I retain totally arbitrary the conclusion that an ACL lesion or insufficiency may be inferred from a lack of passive knee extension, that we can notice in other articular pathologies, such as meniscal tears, loose bodies, etc, that may induce a mechanical or reflex limitation of extension.

But it is quite true and verified by my personal and other authors’ experience that the mentioned maneuver, the extension test, in the presence of an ACL lesion, reveals a unilateral recurvatum [2–6], not specific to that lesion, that must be validated by the Lachman test and by functional testing.

Should it be disputed that the recurvatum could be attributed to a concomitant postero-lateral rotator instability that is also examined with the external-rotation (E. R.) recurvatum test of Hughston, it may be demonstrated by lifting the extremity by the great toe, thus causing excess external rotation and recurvatum which appears as increased tibia vara.

This test is quite different from true recurvatum in neutral rotation, which I am referring to.

In view of the sharp contrast of views on the matter between article authors and me, I retain that my observation might be useful to launch a debate among the Journal readers.
